# Supplementary material for: Exploring the Experience of Breathlessness with the Common-Sense Model of Self-Regulation (CSM)
Source: Healthcare (Basel). 2023 Jun 8;11(12):1686. doi: 10.3390/healthcare11121686 (PMC10298602; doi:10.3390/healthcare11121686)
Supplement: Supplementary file 1 [file healthcare-11-01686-s001.zip › healthcare-2391255-supplementary/Supplemental Materials/Johnston et al CSM and breathlessness.pdf]

Table S1. Interview schedule

Standard question	Alternative or prompt	CSM Components
1. Since we don't know each other, can you tell me about yourself?	How would you describe yourself?	**Identity**
Ok, for these next questions I'm going to get you to think about breathlessness.		
2. Is there anything you have stopped doing or have given up because of breathlessness?	Has breathless changed anything in your day-to-day life?	**Consequences**
3. Can you talk me though … and how breathlessness changed that for you?		
4. Ok still thinking about when you are breathless, what sorts of things help?	Is there anything you do when you are breathless to make it better?	**Control**
5. That's interesting, can you tell me why that helps?		
6. Ok I think I have a good idea of what helps when you are breathless, can you tell me anything that doesn't help when you are breathless?	Are there things that you have tried but don't help when you are breathless?	
7. Still thinking about when you are breathless, what sorts of things are going through your mind?	What sorts of things do you say to yourself when you are breathless?	**Emotions**
8. Can you tell me what kind of feelings you have when you are breathless?	I'm trying to understand what it feels like when you are breathless, can you help me understand what kind of feelings you have when you are breathless?	
Ok now I'm going to get you to think about what you think is going on inside you when you are breathless?		
9. I'm really interested to know what you think is going on inside you when you are breathless… Can you talk me through what you think is going on in your body when you are breathless?	So, what do you think it causing your breathlessness? *Nb – for responses of 'risk' (i.e smoking) or medical condition (COPD) need to explore what they think is going on inside them to cause breathlessness* *Can you talk me through how smoking /COPD is causing your breathlessness? i.e what's going on in your body?*	**Cause**
Just to make sure I have understood, I think you are telling me that you think … is what is going on in your body or causing your breathlessness? Have I got that right?		
10. I'm interested in how you explain your breathlessness to other people like family of friends. Can you talk me through what you usually say to people about why you are breathless?	Can you tell me what you usually say to family or friends if they ask you why you are breathless?	**Identity**
Ok, I'm now going to get you to think about where your understanding of breathlessness comes from.		
11. Where do you think your understanding of breathlessness comes from?	Do you ever look for information about breathlessness? This could be from other health professionals, or books, or the internet?	**Situational stimuli** (information sources)
12. For you, what's been the best or most useful source of information on breathlessness?		
Ok, now I'm going to get you to think about how people have explained your breathlessness to you.		
13. Can you remember anyone talking to you about why you are breathless? Can you remember what they said about why you are breathless?	*Clarify who the person was (Dr, Physio etc.)* What did they say about what's causing your breathlessness?	**Coherence**
14. Did this make sense to you?	*Clarify what made sense and what did not.*	
15. Can you remember them saying anything about what would help your breathlessness?	*Clarify what made sense and what did not.*	
16. Did this explanation change anything about the way you did things?		
Ok, now I'm going to get you to think back to when you first noticed your breathlessness.		
17. How long do you think you have been living with breathlessness?	Can you remember the first time you noticed being breathless?	**Timeline**
18. Can you talk me through how your breathlessness has changed since then?	Do you think this has changed over time?	
19. Do you think this will change in the future?	Can you talk me though how you think your breathlessness might change in the future?	
Ok, these last few questions are about the most helpful and not helpful things people have said to you about breathlessness. You may have touched on these before but just to make sure I have understood.		
20. What is the best or most helpful thing anyone has said to you about your breathlessness?	*Clarify WHO and why it was helpful*	**Situational stimuli**
21. What is the worst or most unhelpful thing anyone has said to you about breathlessness?	*Clarify WHO and why it was NOT helpful*	
And the very last question I have is about words. Some people find particular words or phrases positive, comforting or supportive.		
22. Are there any words or phrases that you like or prefer when talking about your health problems and especially breathlessness?		**Situational stimuli**
23. Are there any words or phrases that you really don't like when talking about your health problems and especially breathlessness?		

Table S2: Categorisation of the **‘identity’** domain of the Common-Sense Model of Self-Regulation (CSM)

Identity			
**Meaning unit example**	**Line-by-line categories (inductive)**	**Descriptive categories (inductive)**	**Analytical category (inductive)**
“…at the end of the race I could hardly breathe …” [P: 2030]	Running and gasping for breath	Describing breathlessness	Identity of breathlessness as a symptom
“…oh I can’t really describe it other than that … I guess if you put … a gauze over your mouth …” [P: 2050]	Breathing through gauze		
I: “… explain or describe your breathlessness to a family or friend ?”<br>P: “… if you laid down on the floor and I … put weight on your chest.” [P: 2050]	Weight on your chest		
“It’s [breathlessness] like be[ing] strangled.” [P: 2050]	Strangulation		
“Yeah, [breathlessness is] like drowning almost.” [P: 2050]	Drowning		
“… I know I had to fight it [breathlessness] on my own and it’s … not nice.” [P: 2060]	Fighting		
“Well I think that my lungs are gonna close …” [P: 2060]	Lungs are closing		
“…they’re [lungs] supposed to be like a balloon … they’ve gone flat.” [P: 3010]	Flat balloon		
“… it [breathlessness] started going downhill.” [P: 2030]	Going downhill		
“I do say sometimes I wish I could put my hand down there and get out whatever it is that’s stopping me from breathing.” [P: 2030]	Something you want to get out		
"I walk to the park and huff and puff a bit and huff and puff a bit back …" [P: 2010]	Puff and huff		
“…in the beginning I just thought it was a bit of a nuisance.” [P: 2090]	Nuisance		
“Because I don’t think I’m really bad. Like I’m not really bad.” [P: 1010]	Not bad		
"Now I don’t know whether it’s [breathlessness] just habit or not? " [P: 2010]	Just habit		
“you almost get the feel that they’re saying ‘oh you’re just overweight and unfit’.” [P: 2090]	Misunderstood	Breathlessness isn’t an experience others can relate to	
“ … people see me and I … suspect they’re thinking ‘what’s the matter with him? What a lot of bullshit, he’s fine?’” [P: 2050]	Invisible		
“…you’re breathless… it’s not ah something that’s very explainable…” [P: 2090]	Unexplainable		
“With this [breathlessness] I certainly feel alone.” [P: 2090]	Isolating		
“…I’m a mum of six kids. … I have some health problems. … have some breathing disorders and stuff and a fulltime carer for two of my kids.” [P: 3060]	Breathlessness mentioned in relation to identity	Breathlessness as part of my identity	
“. … I’m 78, … I’ve got a … undergraduate degree in physics and geology and a Masters degree in geophysical exploration, which I … studied in America, got a scholarship over there. … worked in the oil and gas business...” [P: 2040]	Breathlessness not mentioned in relation to identity		

Table S3: Categorisation of the **'control'** domain of the Common-Sense Model of Self-Regulation (CSM)

Control			
**Meaning unit example**	**Line-by-line categories (inductive)**	**Descriptive categories (inductive)**	**Analytical categories (inductive)**
"…I do breathing exercises … And this is where [respiratory physiotherapist] … has been teaching me to do and I find that very helpful." [P: 2010]	Breathing exercises	Physiotherapy-led strategies	What I have learnt to do when I'm breathless
"…with [respiratory physiotherapist's name] help I could feel my breathing getting better … with the machine she gave me to … do the exercises …" [P: 3080]	Physiotherapy		
"[Puffs through mouthpiece] So I'm coughing up a bit of mucous, which hopefully's helping …" [P: 2040]	Airways clearance		
"…sometimes it gets me so exhausted I have to go on the CPAP afterwards [after showering]." [P: 4010]	CPAP (continuous positive airway pressure)	Equipment	
"I use that inside the house [touches four-wheeled walker] because … it's easy to walk." [P: 2070]	Walking aids		
"…it's a lot better now because I'm on … medication …" [P: 2020]	Medication	Prescribed by a doctor/specialist	What the doctor told me to take when I'm breathless
"I came and doctor [doctor's surname], he was my specialist, arranged for this oxygenator to be installed and for me to go onto oxygen 24 hours a day and … since that time I haven't had those … huge coughing times." [P: 2070]	Oxygen		
"… I've learnt to stop before … it gets scary… So I think I manage it ok. I know when I'm … pushing it too hard so I stop and … catch my breath." [P: 2050]	In control	The amount of control I have over my breathlessness	What I do when I'm breathless
"… I just go to my puffer and I sit and I think 'come on … don't let this take over', but it … does take over really." [P: 2060]	Takes over		
"… you have to say to yourself 'stop it, relax and breathe properly' and that probably helps…" [P: 2010]	Self-talk		
"…the wind seems to take my breath away…" [P: 3020]	Avoiding triggers		
"Well I just sit and try to relax [when breathless]." [P: 3070]	Relax	Relaxation techniques	
"I'm very fortunate I like reading books … that suits someone with pulmonary fibrosis …You can sit down and put your legs up and you … read." [P: 2070]	Distraction		
"I've learnt medical hypnosis … helps to relieve … fear or … anxiety." [P: 1020]	Medical hypnosis		
"You just … have to stop what you're doing. That's the only way to help it." [P: 1010]	Stopping	Not overdoing it	
"I've learnt to take my time, to slow down, take my time when I'm doing something and just do it in stages and I eventually I get the job done." [P: 3020]	Pacing		
"… when I've had a bad [asthma] attack I'll go in and just get in the shower and have … a hot steam and that sort of helps to open up … my airways." [P: 3050]	Steam	Opening up my airways	
"I: "…and you think they [exercises] were helping your breathlessness? "<br>P: "I do believe they were … because having not continued with them and Tai Chi the other night was difficult I felt like sitting." [P: 2090]	Exercise	Whole body exercise	

Table S4a: Categorisation of the **'coherence'** domain of the Common-Sense Model of Self-Regulation (CSM) ('it **all makes sense** to me')

Coherence – 'it **all makes sense** to me'			
**Meaning unit example**	**Line-by-line categories (inductive)**	**Descriptive categories (inductive)**	**Analytical categories (inductive)**
I: "So where do you think your understanding of breathlessness has come from?"<br>P: "Oh probably just through me." [P: 3070]	Based on life experiences	Lived experience trumps other information sources	It all makes sense to me
"… I reckon I've got a very good understanding of that [breathlessness]. … I can still take in heaps of air. I've still got a good lung capacity but unfortunately I've killed all the alveoli that processes the oxygen… [P: 2050]	Demonstrates understanding	Understanding demonstrated by clear and confident explanations	
"You've gotta be careful with Doctor Google, … he's not always right … I'll check some of the recent stuff I've done when I go and … see [respirologist] …" [P: 2050]	Cross-checking information	Questions information sources to ensure understanding	
"…if I don't understand I just ask … they [doctors] explain … in my language, … and I do understand." [P: 2030]	Asks questions		
"… [Parkinson's group sessions] and you're able to relate … to what they're saying." [P: 3030]	Relates to information received	Information received is relevant to my experience	
"… when they're [specialist] … explaining it [lung disease] I already knew, it kind of it all made sense to me. It was good to have a figure put on it, 40 percent, 'cause … I thought well that … explains things … " [P: 2050]	Lung function test results equal how my breathing is		
"I've been going to specialists who just confirm that the alveoli between the oxygen exchange, … they're fibrosing, they're dying…" [P: 2090]	Understanding comes from specialist	Understanding comes from health professionals	
"…my understanding is just putting together [respiratory physiotherapist] and [respiratory specialist] … and I've got a GP [general practitioner] …" [P: 2090]	Based on information from multiple sources		
"…I've been going to the same doctors for a long time so they know what is good for me …" [P: 2030]	Confidence in health professionals		
"…if there was a puzzle in my mind … they've [health professionals] kind of helped put it together." [P: 2050]	Putting the puzzle together in my mind		
P: "He [doctor] just says my lungs …. are just ruined, you know, just bad".<br>I: "So he uses pretty harsh words like that?"<br>P: "Yep he did and … I just said 'yeah I know…' [P: 2060]	Agrees with doctor's diagnosis		
"… like doctor says my lungs are severely damaged and there's no, you can do anything about that …" [P: 2060]	Own beliefs influenced by doctor		
"… I do what he [doctor] wants me to do…" [P: 2060]	Does what the doctor says		

Table S4b: Categorisation of the '**coherence**' domain of the Common-Sense Model of Self-Regulation (CSM) ('it **doesn't** make any sense to me')

Coherence – 'it **doesn't** make any sense to me'			
**Meaning unit example**	**Line-by-line categories (inductive)**	**Descriptive categories (inductive)**	**Analytical categories (inductive)**
"… whatever's gonna happen gonna happen…" [P: 2040]	Luck	I don't understand what my sources of information tell me	It doesn't make any sense to me
"In the early days I couldn't understand because it looked like I would be living … the rest of … my life … coughing." [P: 2070]	Lack of understanding initially		
I: "… what do you think is going on inside your lungs?"<br>P: "Wouldn't have a clue." [P: 3010]	Uncertain		
"… I went to the doctor the next morning and she said 'why didn't you call an ambulance you idiot?' 'Cause I'd lost quite a bit of blood…" [P: 1010]	Failure to interpret severity of symptoms and act appropriately		
"…whether or not the rattles are part of the breathlessness specifically or the wheeze I really don't know." [P: 1020]	Doesn't understand doctor's explanation		
"…home oxygen monitoring … Whether or not that's got anything to do with breathlessness I've got no idea." [P: 1020]	Doesn't understand test results		
"… they [doctors] said … there's no cure I couldn't understand that…." [P: 2070]	Hard to understand that they say there's no cure/they couldn't stop it		
"… the doctor's jargon … Some of it you have to get them to explain down to normal terminology…" [P: 3060]	Jargon is confusing		
"… it seems to have stabilised, but … according to my specialist I've got … 40 percent lung capacity for a person my age." [P: 2050]	Lung function test results/medical opinions do not match how I feel	I disagree with what my information sources tell me	
"You read the internet and you think your gonna die in a couple of years, but they [doctors] said no not yet." [P: 2070]	Internet gives incoherent information		
"… they said 'oh it won't get any worse if you look after yourself', but that's not true I don't think." [P: 2050]	They say it won't get worse but I think it will		
"The doctor says if it's windy don't go out, … if it's really cold stay inside … and I feel I've lost a quality of life." [P: 2060]	Doctor's advice is incoherent with desires		
"… the smoking like the doctor says has … not done me any good but … the real cause was I had a weak chest…" [P: 2060]	Own beliefs outweigh doctor's beliefs		
"… [doctors] just say 'oh that's nothing'. Whereas I'm thinking oh that's the bronchiectasis getting worse, but it probably is nothing [laughs]." [P: 1010]	Doctor brushes off symptoms that concern the individual		
"I honestly thought … she'll … give me a tablet and I'll go home and it'll be alright. … I'm pretty sure she indicated that it would probably get worse…" [P: 1010]	Individual's beliefs not the same as doctor's opinion		
"There's an ad on TV tryin' to get you to get off smoking ... I don't believe it and never will." [P: 3010]	Disagrees with media depiction of breathlessness		
"… I have no idea if that's a fact because I don't really know. You see I'm not one who reads up on stuff." [P: 1010]	Demonstrates lack of understanding	Demonstrated by inability answer question	It doesn't make any sense to me
I: "… the things you read online do the all make sense?"<br>P: "Sometimes, sometimes not…" [P: 3060]	Information sources conflict with one another	Confusion arises when information sources conflict with one another	
"I understand it but it doesn't make sense. … I understand what the collapsing of the trachea looks like in my head but I don't know why…" [P: 4010]	I understand, but that doesn't mean it makes sense	Understanding doesn't answer all my questions	
"… I think that a lot of medical teams need to actually realise … that although someone might be displaying extreme anxiety … maybe they're anxious 'cause they can't breathe." [P: 4010]	Assumptions made by health professionals do not match individual's beliefs	Health professionals make assumptions that don't align with the individual's beliefs	
