# Supplementary material for: Exploring the Experience of Breathlessness with the Common-Sense Model of Self-Regulation (CSM)
Source: Healthcare (Basel). 2023 Jun 8;11(12):1686. doi: 10.3390/healthcare11121686 (PMC10298602; doi:10.3390/healthcare11121686)
Supplement: Supplementary file 1 [file healthcare-11-01686-s001.zip › healthcare-2391255-supplementary/Supplemental Materials/Supplemental Materials Methodological details framed by COREQ_checked.pdf]

Supplemental Material:

Methodological details framed by COREQ (COnsolidated criteria for REporting Qualitative research) Checklist

**Domain 1: Research team and reflexivity**	
Personal characteristics	
1: Interviewer/facilitator	Rebecca Burgess [RB]
2: Credentials	Bachelor of Physiotherapy (Honours) and was enrolled in Masters by Research.
3: Occupation at time of the study	At the time of the study, RB was a research degree candidate [0.8EFT] and employed fractional (0.2EFT) as a physiotherapist
4: Gender	Female
5: Experience and training	In preparation for the conduct of this study, RB completed a university short course in qualitative research. Prior to conduct of this study RB was mentored (including super-vised practice and feedback) to develop qualitative research skills in participant recruitment, interview technique, interview transcription and data analysis by co-researchers (MW, SK and KJ) who all have experience and prior publications in qualitative research.
Relationship with participants	
6: Relationship established	RB (and members of the research team, MTW, KNJ, SK) had no prior relationship with participants prior to study commencement.
7: Participant knowledge of the interviewer	Participants had no prior knowledge of the interviewer
8: Interviewer characteristics	RB is a Generation Z (born between 1995 and 2010) graduate physiotherapist who was undertaking the study as part of a Masters<br>by Research supervised by three experienced researchers (MTW, KNJ, SK), two of whom were physiotherapists (MTW & KNJ). RB had some prior knowledge and working relationships with people experiencing breathlessness in daily life from undergraduate physiotherapy placements, part-time employment in residential aged care and family/friends. RB commenced working part-time as a physiotherapist in residential aged care at the same time as commencing this study and continued to work with people experiencing chronic breathlessness in this role. At the commencement of the study, based on her prior experiences, RB believed breathlessness was a sign of worsening chronic disease and a precursor to death. Attending the 2018 Dyspnea Society international scientific meeting and the Practical Management of Chronic Breathlessness course (Johnston et al. 2020) widened the lead researcher's knowledge of breathlessness and informed her of its multidimensional nature.
**Domain 2: Study design**	
Theoretical framework	
9: Methodological orientation and Theory	Generic descriptive qualitative approach with content analysis framed by Common Sense model of Self-Regulation
Participant selection	
10: Sampling	People were eligible for inclusion if they were: 1) aged over 18 years; 2) experienced breathlessness in daily life; 3) receiving medical management for the underlying cause of breathlessness; 4) able to comprehend written and spoken English; and 5) able to give in-formed consent.<br>Purposive recruitment sought to reflect diversity (maximum variation) in the impact severity of exertional breathlessness by aiming to include at least one participant with each of the five grades of the modified Medical Research Council dyspnea scale (mMRC). The mMRC grades range from 0 ('only get breathlessness with strenuous exercise') to 4 ('too breathless to leave the house or when dressing/undressing').<br>Participants were recruited from either: 1) physiotherapy practices in metropolitan Adelaide, South Australia; 2) people who had completed the Community Breathlessness Intervention Service (BLIS) program pilot study; or 3) a University of South Australia health clinic
11: Method of approach	In response to information flyers available in each recruitment site, potential participants contacted the research team to express interest or seek further information about the study. All potential participants were screened in-person or via phone or email to determine their eligibility for inclusion.

	Where participants met the inclusion criteria and indicated a willingness to participate in this study, a mutually convenient time was arranged for the study interview at either their home or at the University of South Australia City East Campus. Participants were provided with honoraria ($50 gift voucher) to recompense for their time.<br><br>Note: The time [days/weeks) between the screening component [mMRC used to assess eligibility) and the scheduled study visit (mMRC repeated) varied, and it was possible that the mMRC grades volunteered on the two occasions may not have been consistent within individuals. The mMRC grade reported at the study session was retained for use in descriptive analysis
12: Sample size	Please refer to item 22 (Data saturation) as these points are reported together
13: Non-participation	Data collection occurred between May and September 2019. Twenty-two participants were screened for this study with 21 participants included in the final analysis (one participant declined due to family illness). Participant characteristics are presented in Table 1. This group included seven men and 14 (66.6%) women (mean age 70 standard deviation (SD) 11) and included at least one participant for each level of exertional breathless-ness severity of the mMRC (range 0 to 4).
**Setting**	
14: Setting of data collection	The semi-structured interview was conducted in a quiet, private room at either the participants home or at the University of South Australia City East Campus, Adelaide, South Australia.
15: Presence of nonparticipants	No other persons were present at the interview besides the participant and interviewer.
16: Description of sample	Participant characteristics are presented in Table 1. This group included seven men and 14 (66.6%) women (mean age 70 standard deviation (SD) 11) and included at least one participant for each level of exertional breathlessness severity of the mMRC (range 0 to 4). Scores for the B-IPQ reflected a range of experiences for the severity of threat posed by breathlessness (total score mean 38.6 SD 9.6, range 13 to 56). There were relatively equal proportions across participants for educational attainment (primary school or lower 24%; high school 24%, certificate or diploma 295, Bachelor's degree or higher 24%). Most participants were living with at least one other person (dual or greater occupancy 66%) and were retired from paid employment (81%).
**Data collection**	
17: Interview guide	To explore participants' experience of breathlessness, a semi-structured interview schedule was developed by the research team (RB, MTW, KNJ, SK) informed by components of the CSM (situational stimuli/information sources /language preferences, cognitive and emotional illness representations) (Appendix Table S1).<br><br>Two practice interviews (RB) including processes for recording, transcription, coding, and synthesis were undertaken with study-naïve colleagues. Recordings and coding were reviewed by the research team (MTW, KJN, SK). Feedback provided concerning rapport building, verbal and nonverbal communication, question wording, use of standardized prompt questions, timing and flow of questions were incorporated in subsequent practice. Recruitment and data collection processes were piloted with the first two consecutive participants recruited to the study. Following the pilot processes, no changes were made to the interview schedule. As no changes were suggested or indicated for recruitment, inter-view schedule or data collection processes, data from the first two participants recruited to the study were included within the final analyzed sample.<br><br>All interviews began with question 1 (Since we don't know each other, can you tell me about yourself?) with remaining questions asked in different orders depending on the direction of the conversation and the participant's responses. In the event of distress, participants were given the option of ending the interviewing or taking a break and continuing once they felt able.
18: Repeat interviews	Participants were interviewed once (no repeat interviews)
19: Audio/visual recording	All interviews were video- or audio-recorded as per participant preference.
20: Field notes	Limited field notes were made by the interviewer during the interviews. During the transcription process, comments were added to each transcript regarding non-verbal

	communication as appropriate (e.g., coughs, laughter and the participant showing a prop or making a hand gesture).
21: Duration	For each interview a consistent member of the research team (RB) and participant met for a single study visit of duration up to one hour.
22: Data saturation<br>(Was data saturation discussed?)	There is no standardized method for estimating sample size for qualitative studies [Malterud et al 2016] and the widely used term 'saturation' is increasingly contested [Braun and Clarke 2021].<br><br>The ongoing published critical conversations concerning definitions of 'saturation" (relating to data-, thematic- or code, or meaning-saturation) and whether this concept is always an appropriate and essential requirement of all qualitative approaches /studies was eloquently summed up by Braun and Clarke (2021) as a myth that data saturation is a '…vital rationale and practice for qualitative research more generally" (pg 212). While Braun and Clarke's (2021) commentary focussed upon thematic analysis in qualitative synthesis, these authors concluded that 1) saturation is not always a useful concept for sample size estimates; 2) data saturation is appropriate for some types of thematic analysis (TA) but not others (reflexive TA) and 3) the concept of information power might be more useful for thinking about sample estimates.<br><br>Our prospective planned study and synthesis focused upon descriptive content analysis (rather than thematic analysis) with the intent of identifying content (words/phrases = meaning unit) relevant to components of the CSM (pre-existing theoretical framework, staying as close to the volunteered words /phrases as possible with limited abstraction). With this in mind, we pragmatically estimated our target a priori sample size (minimum of n=20 completed interviews) on the principles of data adequacy (volume, variety and time requirements) rather than principles of 'saturation' (data, code or 'theme'). In terms of data volume, our final interview schedule included 23 questions, 22 of which were eligible for inclusion in content analysis targeting specific CSM components or language preferences for breathlessness. In terms of variety, we ensured that our sampling included people with varying degrees of breathlessness impairment (mMRC grades). We anticipated that, for each interview, there would be 10 to 20 hours of transcription and analysis). We reviewed sample estimates for similar study approaches using the CSM and elected to use the larger sample range.<br><br>Data adequacy: In terms of whether our study had sufficient data to be confident in our higher order analytic categories.<br><br>• Within the first interview, transcript text was extracted that referred to all six cognitive representations described by the CSM, emotions, and information sources about breathlessness, indicating an immediate good fit with the CSM.<br>• After the 4th interview, 12 analytical categories (for cognitive and emotional representations) and all categories for information sources were represented in the data.<br>• After the 7th interview, seventeen analytical categories had been derived<br>• After analysis associated with the 17th interview, data was adequately representing 19 analytical categories, including both confirmatory and disconfirming evidence (Vasileiou et al 2018).<br><br>• A further four interviews (n=21) were conducted and prospectively analysed: all data aligned with the existing 19 analytical categories associated with CSM domains. We judged that this data was adequate to explore and support applicability of the selected domains of the CSM to the experience of breathlessness.<br><br>While this could be considered a form of reviewing 'data saturation at a code/category level, this was not part of our decision-making process for estimating our sample size or cessation of data collection
23: Transcripts returned	Interview transcripts were emailed or posted to participants for checking no later than one week after the interview. A preliminary analysis of the interview transcript was undertaken (RB) and a list of statements and questions for clarification with the

	participant was compiled. During a follow-up phone call with participants the researcher confirmed the accuracy of the interview transcript, clarified any areas of uncertainty or additional comments, and sought the participant's perspective on the preliminary analysis.
**Domain 3: analysis and findings**	
Data analysis	
24: Number of data coders	Coding and content analysis processes were completed by a consistent member of the research team (RB) within a week of each interview. Every third interview transcript was independently coded by a second member (KNJ). Simultaneous data collection (RB) and analysis (RB, KNJ) continued during this time, at a rate of one to two interviews per week. Coding decisions were reviewed iteratively through meetings between the two team members throughout the data collection process.<br>On completion of data collection and initial coding and content analysis, the research team reviewed all coding, descriptive and analytical categorization for discrepancies or areas of ambiguity. Overlapping categories and meaning units were discussed with the consensus that overlapping categories could be combined and meaning units could belong to more than one category. Consideration was given to plain language category labels and condensing data to facilitate transparent and intuitive reporting.
25: Description of the coding tree	Content analysis was conducted based on the methodology outlined by Elo and Kangäs (2008), documented within a spreadsheet (Microsoft Excel, Microsoft Corpo-ration 2010 version 14.7.3).<br><br>For cognitive and illness representations of the CSM specific to the experience of breathlessness:<br>'Meaning units' were defined as specific words, sentences or paragraphs that related to one another due to their content or context irrespective of the interview question or where the word/phrase appeared in the transcript [33].<br>Data in each transcript were analyzed using these steps:<br>1) Open coding of meaning units using components of the CSM as an organizing framework (deductive process). During open coding, meaning units were gathered according to the relevant CSM component (i.e. situational stimuli, cognitive and emotional representations.<br>2) Labelling of meaning units and creation of categories within CSM components (inductive process). Using a line-by-line approach, meaning units were labelled (line-by-line categories) and those that shared similar ideas/concepts within each CSM component were grouped together in descriptive categories.<br>3) Abstraction (inductive process). Similar descriptive categories were grouped into higher order analytical categories.<br><br>Meaning units were first identified from the interview transcript (Microsoft Word) and copied to a spreadsheet (Microsoft Excel) where columns reflected components of the CSM (Step 1). Additional sub-columns were created and re-configured (Steps 2 and 3) in subsequent versions of the spreadsheet as data collection and analysis continued (additional row for each participant).<br><br>An overview of higher order /key analytical categories for all cognitive and emotional representations reflecting CSM components are summarized in Table 4. Detailed tables showing all generations of analysis with unedited meaning units, line-by-line categories, descriptive categories and analytical categories are included for cognitive representations of identity, control and coherence in Appendix (S2, S3 and S4a and b).
26: Derivation of themes<br>Were themes identified in advance or derived from the data?	The CSM was prospectively planned as the theoretical framework with 'descriptive' and 'higher order analytical categories' derived from the data (categories rather than themes).<br>Meaning unit data in each transcript were initially coded<br>using components (i.e. situational stimuli, cognitive and emotional representations) of the CSM as an organizing framework (deductive process).<br>Similar 'line-by-line' categories for each CSM component were grouped within a 'descriptive category' (inductive process).<br>Similar descriptive categories were grouped into higher order analytical categories (inductive process).

27: Software	Meaning units were first identified from the interview transcript (Microsoft Word) and copied to a spreadsheet (Microsoft Excel, Microsoft Corporation 2010 version) where columns reflected components of the CSM (Step 1). Additional sub-columns were created and re-configured (Steps 2 and 3) in subsequent versions of the spreadsheet as data collection and analysis continued (additional row for each participant).
28: Participant checking	Participants did not provide feedback on the overall study findings.<br>On completion of the study all participants were provided with a plain English summary of the study findings.

References:

Malterud, K.; Siersma, V.D.; Guassora, A.D. Sample size in qualitative interview studies: guided by information power. Qual Health Res 2016, 26, 1753-1760. DOI: 10.1177/1049732315617444

Braun, V.; Clarke, V. To saturate or not to saturate? Questioning data saturation as a useful concept for thematic analysis and sample-size rationales. Qual Res Sport Exerc Health 2021, 13, 201-216. DOI:10.1080/2159676X.2019.1704846.

Vasileiou, K.; Barnett, J.; Thorpe, S.; Young, T. Characterising and justifying sample size sufficiency in interview-based studies: systematic analysis of qualitative health research over a 15-year period. BMC Med Res Methodol 2018, 18, 148. DOI.org/10.1186/s12874-018-0594-7

Elo, S.; Kyngäs, H. The qualitative content analysis process, J Adv Nurs, 2008, 62, 107–115. DOI: 10.1111/j.1365-2648.2007.04569.x

Johnston KN, Young M, Kay D, Booth S, Spathis A, Williams MT. Attitude change and increased confidence with management of chronic breathlessness following a health professional training workshop: a survey evaluation. BMC Med Educ 20, 90 (2020). https://doi.org/10.1186/s12909-020-02006-7
